# New Tobacco and Tobacco-Related Products: Early Detection of Product Development, Marketing Strategies, and Consumer Interest

**DOI:** 10.2196/publichealth.7359

**Published:** 2018-05-28

**Authors:** Yvonne CM Staal, Suzanne van de Nobelen, Anne Havermans, Reinskje Talhout

**Affiliations:** ^1^ RIVM Centre for Health Protection Bilthoven Netherlands; ^2^ Johnson & Johnson Janssen Vaccines Leiden Netherlands

**Keywords:** noncigarette tobacco products, electronic nicotine delivery systems, public opinion, retrospective studies

## Abstract

**Background:**

A wide variety of new tobacco and tobacco-related products have emerged on the market in recent years.

**Objective:**

To understand their potential implications for public health and to guide tobacco control efforts, we have used an infoveillance approach to identify new tobacco and tobacco-related products.

**Methods:**

Our search for tobacco(-related) products consists of several tailored search profiles using combinations of keywords such as “e-cigarette” and “new” to extract information from almost 9000 preselected sources such as websites of online shops, tobacco manufacturers, and news sites.

**Results:**

Developments in e-cigarette design characteristics show a trend toward customization by possibilities to adjust temperature and airflow, and by the large variety of flavors of e-liquids. Additionally, more e-cigarettes are equipped with personalized accessories, such as mobile phones, applications, and Bluetooth. Waterpipe products follow the trend toward electronic vaping. Various heat-not-burn products were reintroduced to the market.

**Conclusions:**

Our search for tobacco(-related) products was specific and timely, though advances in product development require ongoing optimization of the search strategy. Our results show a trend toward products resembling tobacco cigarettes vaporizers that can be adapted to the consumers’ needs. Our search for tobacco(-related) products could aid in the assessment of the likelihood of new products to gain market share, as a possible health risk or as an indicator for the need on independent and reliable information of the product to the general public.

## Introduction

### Background

A wide variety of new tobacco- and smoking-related products have emerged on the market in recent years. Moreover, tobacco companies will keep developing new products to keep meeting the changing needs of their consumers and fulfill changing regulatory requirements. These new tobacco-related products can quickly gain popularity [1], even before there is sufficient scientific evidence to determine their effects on the user and bystander. For instance, recently products marketed as “harm reduction,” “reduced risk,” or “next generation” products [2-4] were introduced making claims of being up to 90% less harmful than conventional cigarettes. Even though these products seem very attractive to consumers, independent scientific research to support these claims is lacking. In addition, these products may be attractive for smokers, but may also be used by nonsmokers. Besides, regardless of their own harmfulness, popular new products could also serve as a gateway to the use of tobacco or tobacco-related products.

Given their potential harmful health effects, and the possibility that the product serves as a gateway to the use of more harmful products, it is important for national authorities and scientists to closely monitor product development of new tobacco and tobacco-related products. Data on product development, marketing strategies, and consumer interest in new products could help to understand potential implications for public health and guide tobacco control efforts [5]. The World Health Organization recognizes the importance of monitoring the availability and regulation of new tobacco and tobacco-related products entering national and global markets [6].

An important determinant of the popularity of the product is product marketing. For instance, previous research has shown that product marketing plays an important role in the perception of the product for potential users of e-cigarettes [7,8]. Factors that increased the likelihood of potential users to try an e-cigarette were as follows: no health warnings, the presence of flavors, and low or no nicotine content. Similar findings have been reported for waterpipe products [9]. Smokers and recent quitters appear receptive to e-cigarettes when exposed to advertisements of the products [10]. Recently, a whole new possibility for marketing has become available through social media. Retailers can easily advertise and promote their products to a large audience through websites such as twitter and Instagram [11,12]. These marketing aspects of the products themselves and in advertisements can lead to fast increases in the popularity of products and should therefore be considered when evaluating the market developments of new products.

### Objective

Retrospective mapping of media trends can provide valuable insights in the types of tobacco products that were popular in the past and can help predict which products will become popular in the future. This paper describes retrospective trends in product development of e-cigarettes, based on data collected over the period of January 2014 until May 2016. During that period, we have collected information from scientific literature and publicly available sources such as news websites and patent databases, to monitor developments in design characteristics of e-cigarettes and related products such as heat-not-burn products. To this purpose, we used an automated search for emerging tobacco(-related) products, and in parallel, we monitored public interest. We have used an approach that has some characteristics of infoveillance, that is, a type of syndromic surveillance that utilizes Web-based contents, and similar aspects as applied by others in this field, that is, using Google searches, social media content, or information from websites [13,14]. Infoveilance has been used in tobacco research to identify trends or to obtain information on perception or user groups [11,15-17]. We have used this approach to identify products that contained new or unconventional design characteristics, such as technical features and decorative elements, to gain insight in the way these products were marketed.

## Methods

### Product Development

To search websites to monitor product development and marketing strategies, we defined a tailored search profile consisting of specific keywords (eg, “e-cigarette” and “hookah”), which were combined with keywords indicating a development (eg, “innovat*” and “trend*”) in both Dutch and English (Multimedia Appendix 1). We selected these languages as we aimed to identify products that were new or soon to be on the Dutch market. For these products, we expect marketing to be in Dutch or English. This search profile was applied to almost 9000 internet sources, which were searched daily (Multimedia Appendix 2). These sources included national and international news websites, websites of online shops, and websites of tobacco manufacturers and wholesalers. Our search resulted in a list of internet links to websites containing information that met our selected keywords. The information on these websites was reviewed for relevancy to our aims. Irrelevant information, eg, not related to tobacco in any way, was removed. These “false” hits were used to optimize the search algorithm by excluding or adding keywords. The initial focus of our search strategy was the e-cigarette. Nevertheless, we also found messages on product development of related products such as heat-not-burn products. The automated search was subsequently extended to follow developments in different classes of products simultaneously.

When a new type of product was identified in our search, a manual internet search and scientific literature search were performed to obtain more information on specific aspects of this new product. In addition, we used product reviews, consumer specific forums, and other publicly available data such as information provided by producers. Next, a database was created containing all new products identified by our search. Products were included in the database when they were considered to have new or unconventional characteristics. This could be products that were based on a new technology, products with new applications (such as the combination with a mobile phone), products that had a new or alternative use (such as new user groups, specific target groups, unintended use), or products showing an increase in popularity (by the increase in messages in our search).

All selected products were stored in the database along with a link to the website where the particular product was found and the date on which the message was found by our search protocol. The database also included (if available) a description of the product (product type, composition, physical parameters, design features, package) and specific product features such as whether or not it contained nicotine, whether tobacco (if present) was heated or combusted, and the type of organic material to be inhaled (such as tobacco or herbs). In addition, the product’s costs relative to that of other tobacco products and its date of market release were stored. Other product marketing information was included if available, such as data on target groups (eg, young adults), reasons for use, prevalence and patterns of use, including possibilities of combined use with other products, awareness and perception of the public toward the product, and attitudes toward tobacco control policies.

It should be noted that the aim of our search was to detect trends in product development of tobacco-related products. We did not aim to detect every new model of the e-cigarette or to receive all information on a product, but instead focused on new technologies, new appearances or applications, and new properties for the user to be able to follow trends.

### Public Interest

In addition, to obtain information on public interest in product groups or specific products over the course of time or in specific countries, global product information searches performed by Google were analyzed using Google trends. The numbers of searches performed for a specific term are reported in proportion to the total number of searches done on Google in that specific time period. Thus, a declining trend line indicates that the relative popularity of a search term decreases, not necessarily that the total number of searches for that term declines. It has been shown that the information obtained from Google trends matched with actual trends in popularity of e-cigarette types [18].

### E-Cigarettes

#### Trends in Devices and Liquids

The increasing numbers of accessories on the market as well as new vaping products combined with unrelated functions attest to a clear trend toward customization of e-cigarettes. It seems that experienced users like to adopt the e-cigarette to their (inhalation) needs, leading to e-cigarettes with adjusted airflow inlet using atomizer heads with different sized air holes [19]. This is applied in the most recently introduced models, which are activated by a pressure difference when the user inhales from the e-cigarette, avoiding pressing a button to heat the device [20]. Other interesting new e-cigarette-like devices provide a combined function with other electronic products such as a Bluetooth e-cigarette, which combines vaping with listening to music or calling friends [21] and another device can be used both as e-cigarette and mobile phone [22].

Moreover, smartphone applications were introduced that track the number of e-cigarette puffs taken, calculate cost savings and increased life expectancy, and have features such as auto-shut down and password protection safety [23]. In line with this, Phillip Morris has filed a patent for an e-cigarette that is Wi-Fi connected, and thus would be able to connect to other devices [24]. This device could potentially synchronize to a smartphone application that is intended to help people quit smoking, and carefully track their progress. A similar product is the Vaporcade Jupiter, a “cellular vaporizer,” combining a smartphone with an e-cigarette [25]. This allows the user to monitor the e-cigarette use, the e-liquid remaining, and the flavor used.

Next to the expanding technical possibilities of e-cigarettes, the variation in e-liquids was growing. At the time of our search, a wide variety of e-liquids in different flavors was available on the market, such as vanilla, cocoa, cherry, tobacco, and coffee [26]. Other available e-liquids strikingly contained vitamins or cannabis flavors. In addition, specific e-cigarettes (mods) are available that allow for not only liquids but also herbs, oils, or fruits to be vaped. Moreover, dual-function devices handle both concentrates and e-liquids using multiple cartridges. We also found that manufacturers attempted to reduce the formation of formaldehyde and metal substances of vapor by producing an e-liquid in which propylene glycol is replaced by vegetable glycerin [27,28].

Waterpipe products also followed the trend of electronic vaping instead of smoking, with products appearing like the e-hookah and “waterpipe flavored” e-liquids [29-31]. Electronic waterpipe products such as e-hookah or e-shisha are highly similar to electronic cigarettes. For example, the Vitacig is a pen-shaped product that produces vapor containing flavorings and vitamins [32]. Most e-hookahs have the size of an e-cigarette, which allows the user to take the product with them easily. But there are also larger e-hookahs available, which are rechargeable, contain multiple flavor cartridges, and have a large capacity battery which can last up to 1500 puffs. Cartridges available for this e-hookah contain up to 12 ml of e-liquid [30].

#### Marketing, Packaging, and Labeling

To gain market share, products are advertised in multiple ways and on various platforms. On social media, products are advertised and sometimes offered for free such as batteries for e-cigarettes. Alternatively, free shipping or discounts up to 10% are offered for various products. This offering of products with reduced pricing involves dozens of messages monthly. Manufacturer or importer websites sometimes offer the possibility to become an ambassador of their product line [33]. Such an ambassador is expected to set up a community to share experiences and being active as a blogger or on social media. Some marketing activities were specifically aimed at youth by focusing on youth culture. For instance, pop-up bars set at various events and locations are part of the campaign of the e-cigarette “Juul.” The “bar” in this case is a vapor lounge with brightly colored billboard display. In addition, images or cartoons of a young woman were shown in advertisements. Other advertisements were aimed at smokers who would switch from cigarettes to e-cigarettes, describing the technology of some products as “developed by smokers,” “same feeling as a real cigarette,” or “smoking the healthy way.”

General themes that were seen in marketing of e-cigarettes are health, lifestyle, and personalization (Table 1). Wordings implicating the product is a healthier alternative are often used in marketing, such as “natural,” “food- or pharma grade,” “homeopathic,” and “made in Switzerland.” These terms refer to the plastics or e-liquid used or more specifically to the nicotine in the product. However, in most countries, worldwide restrictions on tobacco advertising are active. Before May 20, 2016, European regulations advised “to adopt a restrictive approach to advertising electronic cigarettes and refill containers” [34]. Following the Tobacco Product Directive, which became effective on May 20, 2016, advertisements or promotions for tobacco products, including e-cigarettes, are no longer permitted.

**Table 1:** Product appeal and marketing terms used for online e-cigarette advertisement.

Product name	Marketing terms	Product appearance
E-Njoint	Natural, harmless, safe, cartoon young woman	Bright colors
Juul	Stylish, intensely satisfying, intelligent design, elegant, innovative, young people	Bright colors, design
ExcluCig	Exclusive, luxurious, fashionable, high quality, young woman	—^a^
Treasurer vape	Elegant, discrete, pure, high quality, high-end product	White, light grey, flowers, design
Vaporcade Jupiter	Technology, discrete, quality, young woman	Black, design
Innokin lily	Elegant, luxurious, exclusive, beautiful vaping, young woman, highest quality, design	Swarovski crystals, flower, colors
Zensations	Unique, like real cigarette, variation of tastes	Design
Cig-a-LinQ	A Dutch brand, next generation, developed by smokers	Stylish

^a^Information not available.

**Table 2:** Packaging and labeling information of e-liquids upon online ordering from several Dutch Web shops. Information as visibility of a health warning on the package or on the website as well as nicotine content and age verification are reported. A total number of 25 Web shops were randomly selected; from each Web shop, at least 2 products were assessed. Information was obtained before the Tobacco Products Directive came into force.

Information on packaging or website	Not present (%)	Present (%)
Health warning visible on packaging	84	16
Health warning on website	80	20
Nicotine content indicated	0	100
Age verification	60	40

#### Design and Packaging

The latest type of customizable e-cigarettes with Bluetooth function, colored led lights, and vitamins show that not just the technology is changing toward the user’s needs but also their functionality and appearance.

Stylish packaging, product design, and color choices of the product, such as stainless steel, match marketing terms such as “elegance’ and ‘discrete.” Moreover, the flavors, colors, naming, and electronic capabilities of the products could specifically appeal to young people and unexperienced users. Flavors that are added to e-liquids are often sweet and fruity (eg, apple, melon) or tobacco-like or they give the impression of gaining energy (coffee, energy) or a soothing sense of holiday spirit or relaxation (cocktails, tropical, cannabis). A significant number of Europeans consider a reference to flavors and pack color to be indicative for the level of harm of a tobacco product [35].

#### Health Warnings

Consumers consider health warnings and nicotine levels presented on a product to indicate the level of harm [35]. We assessed the packaging and labeling of 25 randomly selected e-liquids available for sale on Web shops in the Netherlands (Table 2). We found that nicotine content was often indicated in the advertisement text, but health warnings were generally not visible on product photos of the outer package nor on the website of the shop selling them. This is in contradiction to packages of regular cigarettes, almost all of which display a visible health warning on the package. Verification of age of the buyer and information on health effects of the product upon purchase were not clearly present or even absent when buying either tobacco or electronic cigarette products online. In only a quarter of the visited Web shops, an age verification was requested to gain access (Table 2), which can still easily be circumvented by people under 18 years.

#### Public Interest

**Figure 1:**
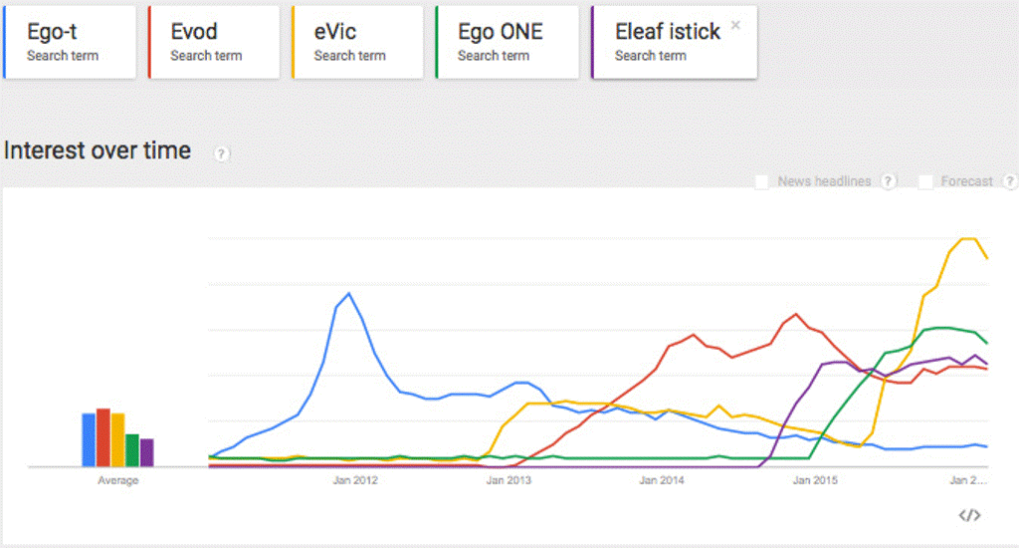
Searches on specific types of e-cigarettes worldwide over time (January 2012 – February 2016) using Google Trends. The interest (y-axis) is shown relative to the total number of searches on Google in this time window.

**Figure 2:**
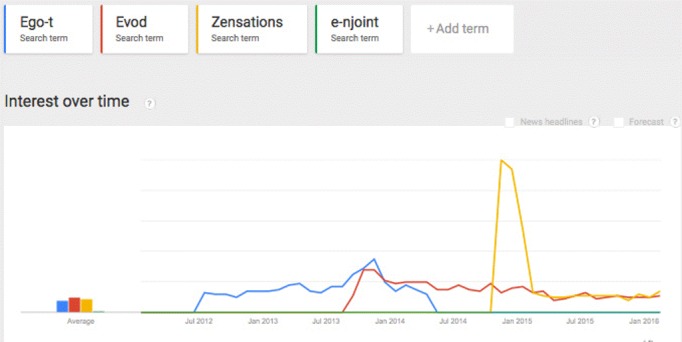
Searches on specific types of e-cigarettes in the Netherlands (1b) over time (January 2012-February 2016) using Google Trends. The interest (y-axis) is shown relative to the total number of searches on Google in this time window.

We analyzed the number of searches on Google to have an indication of the regional interest in different types of e-cigarettes and the changes over time. Public interest in different types of e-cigarettes, such as eGo-, Evod-, and Evic series, are visualized over the past few years in Figures 1 and 2.

These figures show that the number of searches, as an indicator of popularity of a new product, could increase rapidly, but also decline rapidly. The number of searches on products that are available on the market is relatively stable after a while.

### Cigarette-Like Products That Heat Tobacco

#### Trends in Products That Heat Tobacco

Cigarettes that heat tobacco were reintroduced on the market in 2014 by Reynolds (Revo) and Phillip Morris (iQOS). The Revo, by Reynolds, is a cigarette whereby heating is performed using a carbon tip wrapped in glass fibers, which was marketed earlier under the name “Eclipse.” Phillip Morris replaced their formed heatbar (introduced in 2007) by the iQOS, which is a type of electronic cigarette that can heat tobacco. The tobacco product to be heated was to be marketed under the Marlboro brand (currently known as Marlboro heets). Another heat-not-burn device is the Ploom, developed by the Ploom Company and taken over by Japan Tobacco Inc. The Ploom Company, now called PAX Labs Inc, also developed the Pax and the Pax 2. These are both products that vaporize heated tobacco. Furthermore, The Firefly developed The Firefly 2, which heats loose-leaf plant material and concentrates and is often used to vaporize marihuana. During the time of our search, British American Tobacco was developing a product that combines tobacco and e-cigarette technology by heating nicotine-laced liquid into an inhalable vapor that passes through a bit of tobacco near the tip (iFUSE) [36].

#### Marketing, Packaging, and Labeling

Terms used in marketing of cigarette-like products that “heat rather than burn” are referring to the product as “reduced risk” and “innovative.” The appearance of these products is modern and elegant (Table 3).

**Table 3:** Product appeal and marketing terms used for online advertisement of cigarette-like products.

Product name	Marketing terms	Product appeal
iQOS	Reduced risk product, innovative	Clean (white, bright blue), stylish, elegant
Revo	Reduced risk	Similar sized package as traditional cigarette, white or light grey or gold
PAX 2	Smaller, smarter, sleeker	Design, elegant and fun
iFuse	Reduced risk	Packed as traditional cigarette, stylish

**Figure 3:**
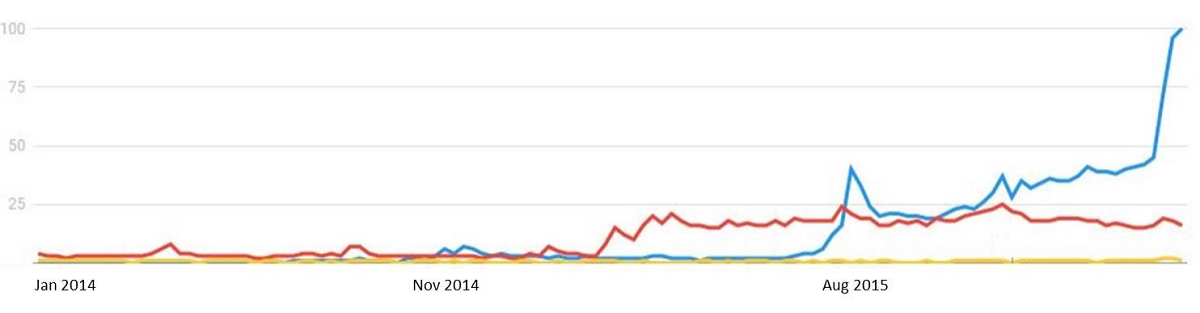
Relative number of searches for heat-not-burn products: iQOS in blue, Pax 2 in red and iFUSE in yellow, worldwide in the period January 2014- May 2016) using Google Trends.

#### Public Interest

Figure 3 shows the relative frequency of internet searches for heat-not-burn products. The iQOS was first released in November 2014 in 2 pilot projects conducted in the cities of Nagoya (Japan) and Milan (Italy) [37]. In September 2015, the availability of the iQOS was expanded in Japan and was introduced in Switzerland. Analysis of worldwide searches on product information only peaks in the 2 countries of market release: Japan and Italy. Along with its market introduction, interest in the product from Swiss citizens was also detected. Most internet searches were performed over time in Japan, indicating the product is gaining popularity there. Most searches on the Pax 2 are conducted in the United States and Canada and seem quite stable over time. In general, and as expected, the relative number of searches and the location of searches coincide with the availability of the product.

## Discussion

### Principal Findings

This paper describes our search for tobacco(-related) products, and, in retrospect, the qualitative data on trends we found over the period January 2014-May 2016. By means of our search strategy, we were able to identify new developments and trends in tobacco-related products and provide information on new products. For example, we identified a trend toward e-cigarette devices that can be more and more adjusted to personal wishes and needs. This was evident from the many available accessories that can be used to adjust physical properties of the e-cigarette, such as airflow. Moreover, our search identified some e-cigarette-like devices with very specific functions, such as playing music or making calls. Additionally, mobile phone applications are available that connect to an e-cigarette and keep track of user behavior. Besides e-cigarettes, our search found that several devices were introduced to the market that heat tobacco instead of burning it. We also found waterpipe products in our search, but we did not elaborate on those in our paper.

Most of the identified new products were marketed using terms indicating less harm and an elegant or luxurious lifestyle. This is in line with an earlier study by Escobedo et al [38], who found that e-cigarette websites were much more likely to feature themes related to harm reduction. In addition, marketing of some products was aimed at specific target groups such as young/unexperienced consumers or smokers who may switch to these new products. Although advertisements or promotions for tobacco products, including e-cigarettes, are currently no longer permitted, manufacturers can still make their products more attractive (for certain groups) by changing their design and appearance. In line with this, the designs of the products in our search were characterized as stylish and modern.

Although we were able to identify products that seemed to be gaining popularity during the time scope of our search, we cannot draw conclusions about the actual use of the products based on our data. That is, the popularity of products in our study was inferred using the trend data from social media, which reflect the public interest in a specific product rather than the actual use of it [39]. However, as this approach has been taken before [26] and provides an indication of the consumer perception [18], this seems to provide a valid estimate of popularity. Although, as e-cigarette proponents appear to be overrepresented on social media [40], this may have biased our findings of the Google trend analysis. We suggest that, when a product appears to increase in popularity based on social media trends, information from other sources (eg, sales data and prevalence data from monitoring studies) should be consulted to confirm whether there is also an actual increase in use.

A limitation of this study is that our approach to our analysis of websites and online retailers could have been more systematic. This study started out as an exploratory search for new products, aiming to find as much information on new products as possible from all thinkable sources. A more structured, systematic approach would have been useful for selecting and classifying relevant information in a clear and objective manner. Previous studies have employed machine learning approaches to classify data from social media [12]. Such approaches seem also suitable for the identification of novel tobacco-related products, as Allem at al identified 2 new e-cigarette devices by means of a social network analyses of Twitter data. Classification of social media data has not only been shown to be valuable in identification of new products but can also be used for surveillance of product use [12]. Moreover, it can be used to identify and classify the sources of social media messages (eg, retailers, product users), to gain more insight in marketing strategies [41]. Related to this, manufacturers and retailers use social media to increase the popularity of a product such as the e-cigarette, for instance by posting specific types of images [42]. They also promote combined use of products, for instance the use of waterpipe with alcoholic beverages [43]. In addition, other platforms have been used to promote e-cigarettes, such as the Pokémon Go game [16]. Alternatively, social media is also used to promote healthier behavior [44], which can influence the public’s opinion on specific tobacco-related products. For instance, the products that were identified in our search were marketed using terms indicating less harm. Systematic analyses of social media data could thus provide valuable insight in current marketing strategies, which in their turn can influence attractiveness and popularity of specific products.

Overall, our search strategy was suitable for our aims, as it identified some interesting developments. It was also specific, as we received little or no messages outside our scope. By verification with messages from other sources, the completeness of the search tool can be checked. Other sources are, for example, information received from newsletters, information from colleagues in the field, and manual internet searches. In addition, search terms or websites were added to optimize the automated search. The data obtained from the search were also timely, as the time between a notification by our search tool and the actual market introduction was short. For example, the introduction of the E-njoint on the Dutch market was identified in our search a few days before it was officially reported by the Netherlands Institute of Mental Health and Addiction (Trimbos Instituut).

### Implications for Policy and Research

The main concerns related to the use of new tobacco products include unknown toxicity, changes in product use behavior, decreased cessation, increased initiation, sustained prevalence of tobacco “dual use,” and public misunderstanding about the actual risk associated with allegedly less hazardous products. In the United States, the Food and Drug Administration is notified of new products or product changes on the US market [45]. Currently, in the European Union, producers are obliged to notify the national government of their intention to market a new product at least 6 months before market introduction. General guidelines for assessing the risks associated with modified tobacco products have been proposed by the Tobacco Product Scientific Advisory Committee and by the Society for Research on Nicotine and Tobacco [6,46]. This notification allows preparation of requirements, if needed, for communication and marketing of such products to avoid misinterpretation of the harmful effects of the product for the general public [47]. As this information is only available nationally and Web shops facilitate the availability of products almost worldwide, it is important to follow developments worldwide. The information from the producer, together with the information from search strategies like ours, can be used to follow a product over time and may be used for law enforcement. The information can be used to assess the likelihood of new products to gain market share, either as a possible health risk for the general population or as an indication for a need for independent and reliable information of such products to the general public.

## Appendix

Multimedia Appendix 1 Keywords.

Multimedia Appendix 2 Websites searched for the selected keywords.
