# Supplementary material for: New Tobacco and Tobacco-Related Products: Early Detection of Product Development, Marketing Strategies, and Consumer Interest
Source: JMIR Public Health Surveill. 2018 May 28;4(2):e55. doi: 10.2196/publichealth.7359 (PMC5996176; doi:10.2196/publichealth.7359)
Supplement: Multimedia Appendix 1 [file publichealth_v4i2e55_app1.pdf]

Multimedia appendix 1: Keywords

Keyword 1	AND Keyword 2	Or Keyword 3	AND NOT Keyword 4
e-cig	new*	marlboro edge	shooting*
e-cigs	novel*	dutch magic	police*
electronic smoking*	innovat*	vortex tabakskop	financial result*
e-sigaret	revolution*	virgin vapor e-liquid	quarterly report*
e-sigaretten	tasty*	hummingbird fuel	
elektronische sigaret*	trend*		
elektrisch roken	latest*		
hookah*	attract*		
e-cigar	next		
electronic cigar*	sensat*		
e-cigars	nieuw*		
e-cigarette	generat*		
e-cigarettes	recent*		
e-sigaar	gezond*		
e-sigaren	nicotin*		
electronic waterpipe*			
elektrische waterpijp*			
sisha*			
e-smoking			
e-roken			
nicotine-laden substance*			
nicotine laden substance*			
