# Supplementary material for: New Tobacco and Tobacco-Related Products: Early Detection of Product Development, Marketing Strategies, and Consumer Interest
Source: JMIR Public Health Surveill. 2018 May 28;4(2):e55. doi: 10.2196/publichealth.7359 (PMC5996176; doi:10.2196/publichealth.7359)
Supplement: Multimedia Appendix 2 [file publichealth_v4i2e55_app2.pdf]

Multimedia appendix 2: Websites searched for the selected keywords

Categories	
Accountancy<br>Agricultural and fishery<br>Aviation<br>Banks and Insurance companies<br>Business services<br>Construction<br>Culture, sports and entertainment<br>Current<br>Economics and Financial<br>Education<br>Energy, Nature and Environment<br>Financial institutions<br>Fiscal<br>Government<br>Health care<br>Hospitality<br>ICT<br>Industry<br>International<br>International Business<br>International news papers and news sites<br>International organisations<br>International press releases<br>IT and Internet<br>Juridical<br>Knowledge management<br>Lease<br>Life and living<br>Market research<br>Marketing and Sales	Media<br>Mediation<br>Minerals<br>National<br>Other services<br>Personalia<br>Philosophy<br>Police<br>Political parties<br>Press release<br>Property<br>Public administration<br>Real Estate<br>Regional<br>Regional sports<br>Science<br>Social security<br>Society<br>Stock<br>Technique<br>Telecom<br>Tourism<br>Town and village<br>Trade and retail<br>Transport and logistics<br>Transport and storage<br>Water management<br>Weblogs<br>Work and Career

Languages	Number of sites
Danish	8
Dutch	6867
English	1679
Finnish	2
French	48
Frisians	1
German	193
Italian	6
Norwegian	3
Polish	2
Spanish	5
Swedisch	5
Total	8819
